# A validated photonumeric scale for infraorbital dark circles and its application in evaluating the efficacy of a cosmetic treatment product in a split‐face randomized clinical trial

**DOI:** 10.1111/ics.12668

**Published:** 2020-12-18

**Authors:** M.M. O’Mahony, C. Sladen, M. Crone, E. Banner, V.L. Newton, A. Allen, M. Bell, I. Marlow, S.F. Acevedo, L.I. Jiang

**Affiliations:** ^1^ Walgreens Boots Alliance Thane Road Nottingham NG90 1BS UK; ^2^ SGS Stephens 1801 N. Glenville Drive Richardson TX 75081 USA

**Keywords:** dark circles, photonumeric scale, clinical evaluation, claim substantiation

## Abstract

**Objective:**

As a result of their complex aetiology and periodicity, dark circles are difficult to characterize and measure, with current assessment techniques relying on specialist equipment, image analysis or proprietary grading scales. There is therefore a need to develop and validate a photonumeric scale for assessing infraorbital dark circles, which can provide an objective and consumer relevant tool for evaluating this condition and the efficacy of treatment products and procedures.

**Methods:**

A panel of expert clinical evaluators reviewed approximately three thousand facial photographs collected over a 5‐year period and selected images representing a dynamic range of dark circles. A 10‐point photonumeric scale was created, with corresponding descriptors and images for each grade of the scale. To rigorously validate the scale, linearity, sensitivity and precision were assessed by colorimetry and in‐clinic evaluation. Reproducibility was assessed photographically with both experienced and inexperienced clinical evaluators, whereas intragrader repeatability was assessed live in‐clinic. The scale was then employed in a split‐face randomized clinical trial on 58 subjects to evaluate the efficacy of a cosmetic treatment product over 8 weeks.

**Results:**

Colour analysis of the images showed the scale was linear, with statistically significant correlations observed when colour data (CIElab; Individual Typology Angle) were plotted against the corresponding grades (*r* > 0.9, *P* < 0.001). Colour difference (Delta E) was calculated between the infraorbital zone and the surrounding skin, and when data were plotted against the grades, a statistically significant correlation was observed (*r* = 0.99, *P* < 0.01). The magnitude of the Delta E suggested that changes in grade are visibly perceptible to the human eye, and therefore, the scale is sensitive and clinically relevant. Intergrader reproducibility showed strong correlation (0.96) and >90% agreement between experienced evaluators, whereas intragrader repeatability assessment showed >90% perfect agreement between grades. Use of this scale in a clinical trial demonstrated the efficacy of a cosmetic product, with a mean statistically significant (*P* < 0.001) decrease in grade of 0.74 compared to baseline, and 0.59 versus the untreated control, after 8 weeks of treatment.

**Conclusion:**

Our photonumeric scale for infraorbital dark circles is sensitive and robust and provides an objective and easy‐to‐use tool to evaluate dark circles and their treatment.

## Graphical abstract

A 10‐point photonumeric scale was created, with corresponding descriptors and images for each grade of the scale. The scale has been rigorously validated and used in a split‐face randomised clinical trial to evaluate the efficacy of a cosmetic treatment product over 8 weeks.

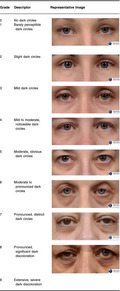

## Résumé

**Objectif:**

En raison de leur étiologie et de leur périodicité complexes, les cernes sont difficiles à caractériser et à mesurer, les techniques d’évaluation actuelles reposant sur des équipements spécialisés, l’analyse d’images ou des échelles de notation exclusives. Il est donc nécessaire de développer et de valider une échelle photonumérique pour évaluer les cernes infraorbitaires, laquelle peut fournir un outil objectif et pertinent pour le consommateur et tester l’efficacité des produits et des procédures de traitement.

**Méthodes:**

Un panel d’évaluateurs cliniques experts a examiné environ trois mille photographies du visage recueillies sur une période de 5 ans, ainsi que des images sélectionnées représentant une plage dynamique de cernes. Une échelle photonumérique à 10 points a été créée, avec des descripteurs et des images correspondants à chaque grade de l’échelle. Afin de valider rigoureusement l’échelle, la linéarité, la sensibilité et la précision ont été évaluées par colorimétrie et en clinique. La reproductibilité a été évaluée sur le plan photographique par des évaluateurs cliniques expérimentés et inexpérimentés, tandis que la répétabilité intragrade a été évaluée en direct en clinique. L’échelle a ensuite été utilisée dans un essai clinique randomisé à deux parties sur 58 sujets, afin d’évaluer l’efficacité d’un produit de traitement cosmétique sur 8 semaines.

**Résultats:**

L’analyse des couleurs des images a montré que l’échelle était linéaire, avec des corrélations statistiquement significatives observées lorsque les données de couleurs (CIElab ; angle de typologie individuel) ont été tracées par rapport aux grades correspondants (r > 0,9, P < 0,001). La différence de couleur (Delta E) a été calculée entre la zone infraorbitaire et la peau environnante, et lorsque les données ont été tracées par rapport aux grades, une corrélation statistiquement significative a été observée (r = 0,99, P < 0,01). L’ampleur du delta E a suggéré que les changements de grade sont visiblement perceptibles à l’œil humain, l’échelle étant par conséquent sensible et cliniquement pertinente. La reproductibilité intergrade a montré une forte corrélation (0,96) et une concordance > 90 % entre les évaluateurs expérimentés, tandis que l’évaluation de la répétabilité intragrade a montré une concordance parfaite > 90 % entre les grades. L’utilisation de cette échelle lors d’un essai clinique a démontré l’efficacité d’un produit cosmétique, avec une diminution moyenne statistiquement significative (P < 0,001) du grade de 0,74 par rapport à la référence, et de 0,59 par rapport au témoin non traité, après 8 semaines de traitement.

**Conclusion:**

Notre échelle photonumérique pour les cernes infraorbitaires est sensible et robuste, fournissant un outil objectif et facile à utiliser afin d’évaluer les cernes et leur traitement.

## Introduction

Infraorbital dark circles is a condition where darkening is observed in the under‐eye area. It is a common aesthetic problem that affects both sexes, a wide range of ages and all ethnicities [1, 2]. The aetiology of infraorbital dark circles is complex; causal factors include excessive pigmentation because of melanin deposition, vasodilation and venous stasis, thinner skin of the eyelids and structural features of the orbital area [3, 4, 5]. This can be compounded by the ageing process, which results in skin sagging and altered subcutaneous fat distribution [1, 2, 3, 4, 5, 6, 7]. In addition, numerous intrinsic and extrinsic factors have been associated with their occurrence [8, 9]. Because of the multifactorial aetiology of dark circles, various treatment strategies and therapies are often required to achieve satisfactory improvements in their appearance [10, 11]. However, to assess treatment efficacy, it must first be possible to accurately evaluate and measure dark circles.

The key principle in the evaluation of dark circles is the assessment of the relative darkness of the under‐eye colour compared to the surrounding facial skin. This can be performed objectively through instrumental measurements or by image analysis [3, 4, 12, 13, 14]. Indeed, these techniques can remove subjectivity and are helpful for showing whether a treatment has induced significant changes. They do however require specialist equipment and rigorous training to perform optimally, especially because of the unique anatomical challenges presented by the location of dark circles in close proximity to the eyes, making many instrumental measurements difficult to obtain. In addition, although instrumental measurement and image analysis are useful for classifying dark circles and understanding causes [4] and may produce statistically significant treatment results, the consumer or patient relevance of these results can be difficult to assess. The ability to provide treatments that patients and consumers can discern is of the utmost importance for customer satisfaction and for those who protect the consumer from misleading claims or false advertising, such as regulatory authorities around the world who review advertising claims in terms of provision of competent and reliable claims that do not mislead, for example the National Advertising Division (NAD) or Federal Trade Commission (FTC) in the United States and the Advertising Standards Authority (ASA) in the UK. These bodies demand that improvements are consumer relevant and therefore discernible and meaningful to members of the public.

Clinical grading scales that require visual assessments are one way to measure changes in skin features and condition in a consumer relevant way. These scales provide descriptors and/or have images to represent and illustrate each point on the scale. Well‐known examples include the Bazin Skin Aging Atlas, Wrinkle Severity Rating Scale and the Griffiths scale for assessing facial photodamage [15, 16, 17]. The 1992 publication by Griffiths et al. used five sets of photographs to illustrate the concept of a nine‐point scale for global facial photodamage, with 0 being no photodamage evident and 8 representing the most severely photodamaged skin, and included descriptors for mild, moderate and severe photodamage. This photonumeric scale concept has been applied to other photoageing parameters, such as crow’s feet wrinkles [18], where grading follows the general guidelines of a modified Griffiths scale. Although dark circles have been evaluated by clinicians’ visual assessments and some proprietary grading scales are available [3, 4, 7, 19, 20], to the best of our knowledge there is currently no published, validated consensus dark circles clinical grading scale, meaning it is not possible to achieve consistency nor directly compare results among laboratories and evaluators across testing centres.

In this study, we set out to develop and rigorously validate a photonumeric scale for the assessment of infraorbital dark circles based on the principles of the Griffiths scale. Following general guidelines for the validation of new techniques [21], linearity, sensitivity and precision were assessed. The scale was then used in a clinical trial, which evaluated the efficacy of a cosmetic treatment product, to demonstrate that the scale is a robust and practical tool for use in the clinic and can be applied to claim substantiation.

## Materials and methods

### Creation of a photonumeric scale for infraorbital dark circles

Approximately three thousand photographs of female subjects, aged 18–75 and of diverse Fitzpatrick skin phototypes, were collected over a 5‐year period as part of the SGS Stephens photo library collection. The photographs were taken using a custom‐designed photo‐station consisting of a Nikon D7000 digital SLR camera (Nikon Corporation, Tokyo, Japan) and unfiltered full‐spectrum light provided by Comet studio strobes affixed to the photo‐station. Frontal view photographs were taken of subjects with their eyes open and neutral facial expressions. As part of the library collection and cataloguing, photographs with dark circles were noted. A panel of experienced clinical evaluators reviewed and selected images that best represented a dynamic range of dark circles. The principles of the Griffiths scale for photoageing [17] were then applied to select scoring grades, descriptors and dark circles images that represented each grade.

### Validation of the photonumeric scale

#### Colour analysis of the scale images to assess linearity and sensitivity

Colour analysis of the images selected for the photonumeric scale was performed using ImagePro Plus software (Media Cybernetics, Rockville, USA). For each image, three areas were selected for measurement: a localized area representing a small square region of interest (ROI) at the inner corner of the left under‐eye area (Inner Corner); a large rectangular ROI covering most of the left under‐eye area (Infraorbital); and a control area below the dark circle on the left cheek bone, representing the surrounding unaffected skin (Cheek Bone). L*, a*, b* (L*a*b* CIELAB 1976; L*, lightness; a*, red‐green component; b*, yellow‐blue component) values were extracted from all ROIs using ImagePro Plus and the ITA (Individual Typology Angle) was calculated as follows [22]:

$${\text{ITA}}^{\circ }=[\arctan(L*‐50)/b*]\times 180/\pi$$

L* and ITA values were adjusted to background skin by subtraction of the L* or ITA value of the inner corner or infraorbital zone ROIs from the L* or ITA value of the cheek bone ROIs. Delta E (ΔE), a parameter that describes colour difference, was calculated using the following formula:

$$\Delta E=\sqrt{{\left(\Delta L*\right)}^{2}+{\left(\Delta a*\right)}^{2}+{\left(\Delta b*\right)}^{2}}$$

Colour differences were determined between the infraorbital and cheek bone ROIs for each image of the photonumeric scale.

#### Evaluator comparisons to assess precision

Reproducibility between four different evaluators (intergrader validation) was assessed by scoring dark circles in a photo deck of 20 photographs. The photographs contained subjects with skin phototypes I–IV and spanned grades 1–8 of the photonumeric scale. Two experienced and two inexperienced evaluators, who had no prior experience of assessing dark circles, each independently scored the 20 images using the photonumeric scale. Intergrader correlation of scoring grades was assessed by calculating Person’s correlation coefficients, with percentage agreement within 1 and 0.5 grades also calculated.

Intraevaluator repeatability was also assessed, but to further test the usability of the scale, the assessment was performed with live subjects in the clinic multiple times. An experienced evaluator used the photonumeric scale to grade 26 subjects (skin phototypes I–V) with dark circles severity grades of 3–6 in a random order at baseline, and again 1 h later. Other subjects were scored between each subject’s initial and validation assessment. After 1 month, 25 subjects were scored in random order, and again 1 h later. Every effort was made to grade the same subjects at baseline and the 1‐month time point. Percentage agreement of exactly matching scoring grades and those within 0.5 were calculated.

### Clinical trial to evaluate the efficacy of a cosmetic treatment product

#### Subject recruitment

Female subjects aged 20–65 with Fitzpatrick skin phototype I–V and mild‐to‐moderate dark circles on both under‐eye areas (grades 3–6 of the photonumeric scale) were recruited to participate in the trial, with the following key inclusion criteria; having a regular and consistent sleep pattern with no planned alteration in sleep pattern during the course of the trial; washout of antiageing products for 4–12 weeks (depending on the product); and no use of foundation or topical eye products 3 days prior to enrolment in the trial. Before participation in any study procedures, subjects were provided with a consent form to read and the opportunity to ask questions about their participation. Written informed consent was subsequently collected from all willing subjects. The trial followed all applicable guidelines for the protection of human subjects as outlined in 21 CFR 50 and in accordance with the accepted standards for Good Clinical Practice (GCP) and the International Conference of Harmonisation (ICH). The trial was conducted at SGS Stephens in Texas (USA) during 2018.

#### Trial design

The clinical trial was an evaluator‐blinded split‐face randomized design. Fifty‐eight female subjects were recruited and provided with an under‐eye dark circles cosmetic treatment product to use twice daily on one randomly assigned eye area, while leaving the other eye untreated. The product was an oil‐in‐water cream, formulated to tackle the key signs of dark circles and contained antioxidant, depigmentation and soothing ingredients. Usage instructions and a diary were provided to each subject and product usage compliance was checked at all time point visits. Subjects were treated over an 8‐week period, with efficacy evaluations conducted by expert grading and photography at baseline, week 1, week 2, week 4 and week 8. Evaluations were performed at the same time of day for each subject. Before each time point, subjects were instructed to remove all makeup at least 30 min prior to each visit and acclimate or at least 30 min in clinic prior to any assessments. An expert evaluator scored the dark circles of each subject’s left and right under‐eye area separately using the dark circles photonumeric scale. Half‐point scoring, where the evaluator deems the observed condition of the dark circle to be between two points of the scale, for example 3.5, was permitted.

#### Digital photography

At each time point, digital photographs were taken using the VISIA CR2 (Canfield Imaging Systems, Fairfield, USA) with a Canon Mark II 5D digital SLR camera (Canon Inc, Tokyo, Japan). Subjects had three sets of full‐face images taken (right side, left side, and centre view) with standardized lighting modes.

#### Statistical analysis

Data collected from all 58 subjects were used for statistical analysis. The mean change from baseline was calculated at each post‐baseline time point, as well as net difference between treated and untreated sites. The Wilcoxon signed rank test was used for treatment comparisons. All statistical tests were 2‐sided at significance level of 0.05. Statistical analyses were performed using SAS software version 9.4 (SAS Statistical Institute, Cary, USA).

## Results

### Creation of a photonumeric scale for dark circles

A photonumeric grading scale was developed as a tool to standardize clinical evaluation of infraorbital dark circles among clinical evaluators and testing laboratories based on the empirical experience of a panel of expert clinical evaluators. This panel of experts reviewed approximately three thousand photographs from a database of facial images and gathered a selection that best represented a good dynamic range of dark circles severity. Based on the principles of the Griffiths scale for photoageing [17], 10 scoring grades (0–9) were selected, each with a descriptor; and dark circles images to represent each grade were finalized by consensus based upon the scale descriptor (Table 1). In addition, the expert evaluators agreed that to achieve maximal accuracy when scoring, both the absolute colour of the infraorbital dark circle and the contrast between the dark circle and its surrounding skin should be considered. No images were selected for grade 0 or grade 9, as no images in the photo library satisfactorily represented these grades and the expert evaluators deemed they were not necessary for full functionality of the scale. This suggested that the two extreme end points are rare in the general population, as no representative examples were found in the extensive photo library of facial images.

**Table 1:** Photonumeric scale for dark circles, with written descriptors and corresponding representative images, compiled from a database of facial images and the consensus opinion of a panel of expert evaluators

Grade	Descriptor	Representative Image
0	No dark circles	
1	Barely perceptible dark circles	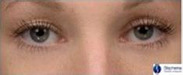
2	Slight dark circles	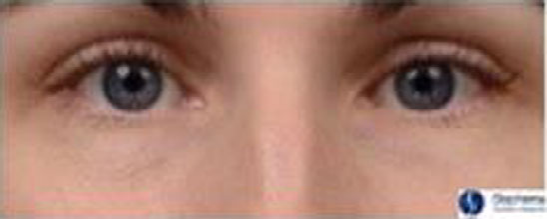
3	Mild dark circles	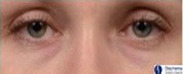
4	Mild to moderate, noticeable dark circles	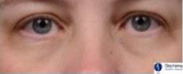
5	Moderate, obvious dark circles	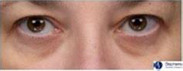
6	Moderate to pronounced dark circles	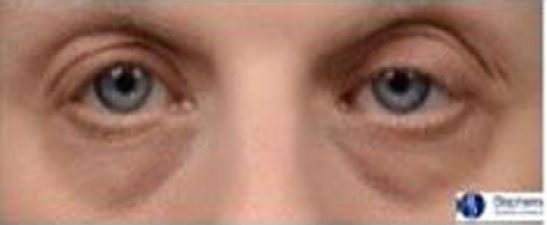
7	Pronounced, distinct dark circles	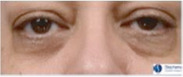
8	Pronounced, significant dark discoloration	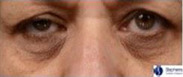
9	Extensive, severe dark discoloration	

### Validation of the photonumeric scale

#### Linearity

**Figure 1:**
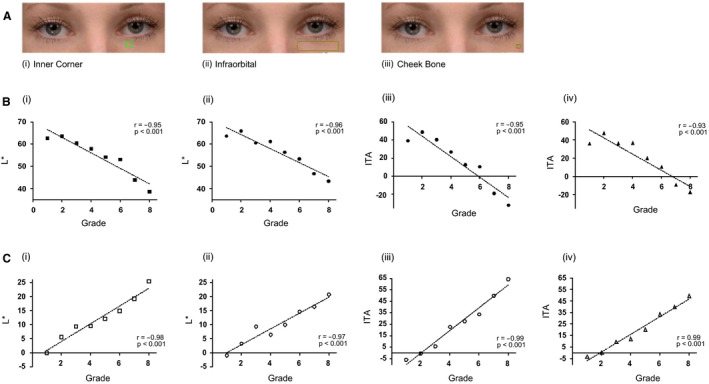
Colour analysis of the selected scale images. (A) The region of interest (ROI) used for image analysis (i) localized area at the inner corner of the left eye—inner corner; (ii) area covering most of the left under‐eye area—infraorbital; (iii) a localized area close to the left cheek bone outside the under‐eye area, representing background skin colour—cheek bone. (B) L* and ITA values from the inner corner (i &amp; iii) and infraorbital (ii &amp; iv) ROIs of the dark circles were compared to grades. (C) L* and ITA values from the inner corner (i &amp; iii) and infraorbital (ii &amp; iv) ROIs of the dark circles adjusted for background skin colour and compared to grades. (*r* = Person’s correlation coefficient; with corresponding*P*values).

To assess the linearity of the scale, image analysis was performed to quantify the colour of the dark circles and its contrast to the surrounding skin on each image of the scale. Three regions of interest (ROIs) were selected for colour measurement on each image (Fig. 1A); a localized area at the inner corner of the left eye (Inner Corner), the infraorbital zone under the left eye (Infraorbital) and a localized area close to the left cheek bone outside the dark circles area (Cheek Bone). L* and ITA values were calculated from each ROI and when the data from the inner corner and infraorbital zone ROIs were plotted against the corresponding clinical grades, strong and statistically significant correlations were observed, with r values ranging from 0.93 to 0.96 and *P* < 0.001 (Fig. 1B). When L* and ITA values were adjusted for surrounding background skin, the correlations with clinical grades showed even better results, with *r* values of 0.97–0.99. The results were again statistically significant with *P* < 0.001 (Fig. 1C).

#### Sensitivity

**Figure 2:**
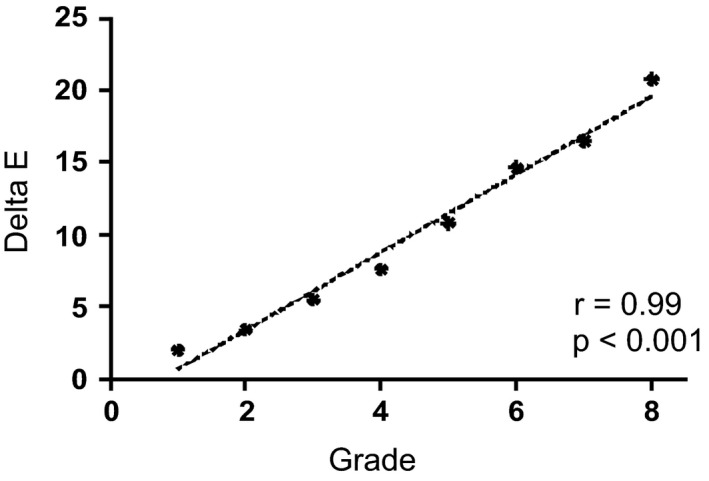
Delta E (ΔE) values, calculated by comparing the colour of the infraorbital and cheek bone ROIs, plotted against grades. (*r* = Person’s correlation coefficient; with corresponding*P*value).

To assess the sensitivity of the scale, Delta E (ΔE) was used to calculate the colour difference between the infraorbital zone and the surrounding skin close to the cheek bone on each image of the scale. When ΔE values were plotted against clinical grades, a strong and statistically significant (*P* < 0.001) correlation was observed, with *r* value of 0.99 (Fig. 2). This was consistent with the L* and ITA analysis. For a clinical grading scale to be successful, it must be sensitive enough to detect changes that are perceivable and meaningful, in this case changes in the colour of the dark circles. Delta E value ranges from 0 to 100 and it is recognized that a ΔE greater than 1 is perceptible to the human eye, particularly for a trained evaluator. Indeed, a ΔE of 1 is often considered the threshold for detection of visible colour change for expert assessment [23, 24, 25, 26]. In addition, some observers have reported that ΔE of 0.5–1 is visible, though this was in a dental setting [27, 28]. Table 2 demonstrates that ΔE values between two consecutive whole‐point clinical grades ranged from 1.41 to 4.26 and are therefore all above the required ΔE of 1 to be perceptible to the human eye. For photonumeric scales, half‐point scoring is often deployed. As the ΔE between the dark circles and the surrounding skin for each grade of the scale was linear (Fig. 2), ΔE was estimated for potential half‐point grades between grades 2 and 6 (Table 3), the range we believe is most prevalent in the general population and appropriate for cosmetic treatment. All half grade ΔE changes between consecutive grades in the range 2–6 were between 1 and 2, which again falls into the range perceivable by human eyes, therefore further confirming the sensitivity and usability of the scale, and the validity of applying half‐point scoring.

**Table 2:** Delta E (ΔE) and difference in ΔE between grades

Grade	ΔE	ΔE difference between grades
1	2.09	‐
2	3.50	1.41
3	5.56	2.06
4	7.67	2.11
5	10.86	3.19
6	14.70	3.84
7	16.55	1.85
8	20.81	4.26

**Table 3:** Delta E (ΔE) and difference in ΔE between half grades

Grade	ΔE	ΔE difference between half grades
2	3.50	‐
2.5	4.53	1.03
3	5.56	1.03
3.5	6.62	1.06
4	7.67	1.05
4.5	9.27	1.60
5	10.86	1.59
5.5	12.78	1.92
6	14.70	1.92

#### Precision

For a photonumeric grading scale to be an objective tool to aid clinical assessment, it needs to be reproducible and repeatable, as well as reliable and easy to use. To assess reproducibility, four evaluators—two experienced and two inexperienced—were instructed to score the same 20 photographs of subjects with dark circles using the photonumeric scale. Grades from the two experienced evaluators set the standard for those images and grades from the inexperienced evaluators were assessed against these grades. In addition, a comparison between experienced and inexperienced evaluators provides insight into the practical usability of the scale when implemented by new clinics. As shown in Table 4, the correlation between the two experienced evaluators was 0.96 with 100% of grades within 1 grade difference, and 90% within 0.5 grade difference. This is considerably higher than the minimum 80% agreement quoted in numerous studies as being clinically acceptable [29, 30, 31]. The two inexperienced evaluators also showed substantial correlation with each other and with both experienced evaluators, with 85–95% of grades falling within 1 grade difference from the other evaluators (Table 4). However, although the Pearson’s correlation coefficients were strong for the inexperienced evaluators, the percentage agreement within 0.5 grade difference was 60–85% and therefore not all assessments met the 80% acceptability threshold. This highlights the need for training of less experienced evaluators to ensure accuracy. Nevertheless, this exercise demonstrates that the photonumeric scale is reproducible and reliable when used among different evaluators. In addition, the scale is easy to use and provides an excellent reference for less experienced evaluators to make consistent assessments, though additional training may be required. As well as assessing results of photo‐grading, intragrader repeatability assessments were performed in‐clinic with live subjects. A fifth experienced evaluator used the photonumeric scale in the clinical trial described below to score the dark circles of 26 subjects at baseline and 25 subjects one month later—22 of the subjects were scored at both time points. At baseline, the evaluator assessed the dark circles of the subjects in random order and again 1 h later. One month later, the evaluator graded the subjects again, twice in random order with a 1‐h interval. At each time point, the evaluator’s grades an hour apart showed 100% agreement within 0.5, with 90–94% of grades being identical (Table 5).

**Table 4:** Interevaluator correlation between experienced (E1 and E2) and inexperienced (IE1 and IE2) evaluators after scoring 20 images using the photonumeric scale as reference

Interevaluator correlation Person Correlation Coefficient					Interevaluator agreement % within 1 grade					Interevaluator agreement % within 0.5 grade				
	E1	E2	IE1	IE2		E1	E2	IE1	IE2		E1	E2	IE1	IE2
E1					E1					E1				
E2	0.96				E2	100%				E2	90%			
IE1	0.91	0.80			IE1	90%	85%			IE1	80%	60%		
IE2	0.91	0.87	0.91		IE2	95%	90%	90%		IE2	85%	65%	60%	

**Table 5:** Intragrader agreement at two time points with a least a 1‐h interval between subjects at each time point (T0; baseline, T1; 1 month later)

T0 v T0 + 1 h		T1 v T1 + 1 h	
% Perfect Agreement	% Agreement within 0.5 grade	% Perfect Agreement	% Agreement within 0.5 grade
90%	100%	94%	100%

### Application of the photonumeric scale in a clinical trial to evaluate the efficacy of a cosmetic treatment product

**Figure 3:**
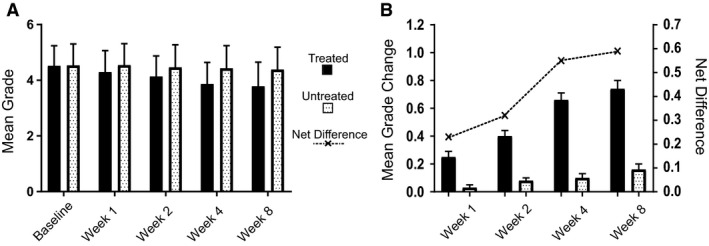
The photonumeric scale was applied in a split‐face randomized clinical trial to evaluate the efficacy of a cosmetic treatment product. (A) Clinical scoring of dark circles in both the treated and untreated under‐eye area over 8 weeks (*n* = 58). (B) Mean grade changes from baseline and net difference in grade treated versus untreated. Improvements were statistically significant (*P* < 0.001, Wilcoxon signed rank test) between post‐baseline grades and baseline grades and between the treated and untreated sites at all post‐baseline time point.

**Figure 4:**
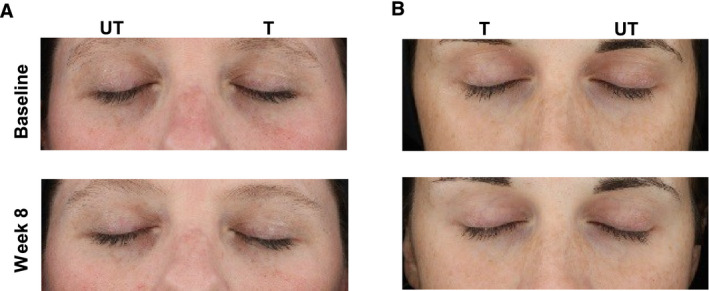
Example images of subjects at baseline and following 8 weeks use of a cosmetic treatment product captured with the VISIA CR2 cross‐polarized light mode. (A) Fitzpatrick skin phototype II, age 36 (B) Fitzpatrick skin phototype II, Age 29. (T = treated; UT = untreated).

The split‐face randomized clinical trial was performed on 58 female subjects with a range of skin types (Table 6). Clinical grades were assessed by an expert evaluator (the 5th evaluator referenced above) at multiple time points over 8 weeks. The results showed a statistically significant (*P* < 0.001) decrease in grade versus baseline for dark circles at each post‐baseline time point representing an improvement in the appearance of the dark circles (Fig. 3A). Additionally, comparisons between the treated and untreated under‐eye areas, based on the mean change from baseline, also indicated a statistically significant improvement in dark circle severity in the treated eyes at each post‐baseline time point, with a steady improvement in the mean grade change and net difference observed as treatment progressed (Fig. 3B). After 8 weeks use of the cosmetic product, 84% of subjects showed an improvement in grade of 0.5 or greater, with a mean grade change of 0.74 compared to baseline. The mean net difference between treated and untreated at 8 weeks was 0.59. The amplitude of both the mean change and mean net difference was more than a half‐point grade and therefore readily visible to the human eye. In addition, over 45% of subjects showed an improvement of 1 grade or more after 8 weeks. Throughout the clinical trial VISIA images were also captured of the subjects. Example images of two subjects are shown in Fig. 4, which visually displays the effect of the cosmetic under‐eye treatment product on the appearance of the dark circles.

**Table 6:** Clinical trial subject demographics

	N	%	Age
**Female**	58	100.0	
**Mean Age**			40.4
Standard deviation			10.7
Minimum			20
Median			39.5
Maximum			61
**Ancestry**			
American or Alaska Native	1	2	
Asian	6	10	
Black or African American	10	17	
White or Caucasian	40	69	
Mixed	1	2	
**Fitzpatrick Skin Phototype**			
I	4	7	
II	17	29	
III	15	26	
IV	12	21	
V	10	17	

## Discussion

Infraorbital dark circles are a common problem, affecting individuals across the globe, and are generally considered aesthetically unpleasing. Many treatments, including topical cosmetic products, have been developed to treat this condition with varying success [1, 2, 10, 11]. Although there are currently methods to assess the severity of dark circles, many rely on specialist equipment or time‐consuming image analysis that requires both specialist skills and software. In addition, some clinics assess dark circles by expert grading of the condition in situ, though these scales vary and are often proprietary information. Therefore, there is a need for a standardized and robust clinical evaluation tool to assess the severity of dark circles and determine efficacy of dark circles treatments objectively and universally.

For any new technique to become established and used as standard, it is first important to address and measure several key criteria, such as linearity, sensitivity and precision. For linearity, the new procedure must obtain results that are directly proportional to true values within a range. For sensitivity, the procedure must be able to record small variations or differences within the range. Precision consists of assessing reproducibility, where different assessors using the procedure, on different days with different subjects, should generate similar results; and repeatability, where the same assessor using the procedure under similar conditions within a short time interval and with the same subjects should also generate similar results [21]. In this work, we have created and validated a new photonumeric grading scale for infraorbital dark circles that covers an excellent dynamic range of severity; and demonstrated that the scale is linear, sensitive, reproducible and repeatable. Furthermore, as approximately three thousand facial photographs were assessed during the development of this scale, we believe the grading scale is representative of the prevalence of the condition in the general population.

Reproducibility was assessed photographically with both experienced and inexperienced clinical evaluators. The results showed excellent reproducibility of scoring, particularly for the experienced evaluators. The two inexperienced evaluators also showed acceptable correlation with each other and with both experienced evaluators, with 85–95% of grades falling within 1 grade difference from the other evaluators. By using inexperienced evaluators, we have demonstrated that the scale is easy‐to‐use, even for non‐experts, though additional training may be necessary for full accuracy, and the scale would be a valuable asset for ensuring consistent in‐clinic assessments of dark circles, as well as being a useful tool for training new evaluators. Intragrader repeatability was assessed in‐clinic with live subjects at a number of time points. The results showed excellent intragrader repeatability, with 100% agreement within a 0.5 grade, and 90–94% of grades being identical. We believe that use of photographic and in‐clinic assessments further confirms the applicability and versatility of this new photonumeric scale.

The images selected for this photonumeric scale that best represented the dynamic range of dark circles severity were mainly of subjects of skin phototype I–III, that is fair skin tone. This raises an important question of how applicable it is for assessing this condition in subjects with darker skin tones. To address this, the photographic reproducibility and in‐clinic repeatability assessments were performed across multiple phototypes to prove its applicability to all skin tones, and excellent results were achieved. Furthermore, when the Delta E colour difference between the dark circles and their surrounding skin was analysed, it showed better correlation with the clinical grades than did the absolute colour of the dark circles. This indicates that colour contrast, as well as absolute colour, is important for expert evaluator scoring of dark circles.

As previously discussed, for a clinical grading scale to be truly successful it must be sensitive enough to detect changes with treatment that are perceivable and meaningful to individuals. We have clearly demonstrated the near‐perfect linearity for colour progression and colour contrast, meaning it is possible to interpolate the midpoint between two whole grades, thereby allowing half‐point scoring, as has been demonstrated in other scales. Image analysis revealed that the ΔE colour difference between most whole grades was approximately 2 or greater, meaning a presumptive change for each half‐point grade difference of 1–2, which is perceptible to the human eye and therefore clinically relevant.

To ensure the photonumeric scale was robust and able to detect treatment changes in a clinic setting, we applied the scale to an evaluator‐blinded split‐face randomized clinical trial of a dark circles cosmetic product. Park et al. previously used their visual grading scale, based on Korean subjects, in a product efficacy trial, but were not able to demonstrate statistically significant differences between the product and a placebo over 8 weeks with the scale, even though they could show significant differences using instrumental and image analysis techniques [7]. Here, we have been able to demonstrate our photonumeric scale can be successfully applied to product efficacy trials and can be applied across diverse phototypes. Using the scale, a mean change in grade of 0.74 was observed in the treated eyes compared to baseline following 8 weeks of product application. Although there was also a slight change in grade in the untreated eyes at 8 weeks, the net difference between treated and untreated was 0.59, and crucially, there was a continuous decrease in the grade over the treatment period. A slight change is the untreated eye is not entirely unexpected, as we have previously observed temporal fluctuations in the appearance of dark circles [3]. Our data have shown that changes in grade of greater than 0.5 correspond to ΔE values of between 1 and 2, which is within the range perceivable to the human eye and therefore the mean treatment change observed in the clinical trial is consumer relevant. Indeed, further interrogation of individual subject’s data revealed that over 45% of subjects had an improvement in grade of 1 or more, which in general corresponds to ΔE values of greater than 2. At this level, colour difference is even more perceptible to the human eye, and a clearly noticeable difference would have been observed in these subjects after 8 weeks of treatment, even to the non‐expert eye. As a steady decrease in grade was observed with treatment, we anticipate that the improvement in the appearance of dark circles observed would continue with prolonged treatment.

Additionally, in the clinical trial conducted, 38% of subjects fell into the darker skin tone category with skin phototype IV or V. When data for dark circles grades were grouped according to skin tone (lighter phototypes I–III—versus phototypes IV–V), the results for the mean change in grade after 8 weeks were similar (0.76 vs. 0.70), confirming the product is effective for multiple skin tones and treatment efficacy can be assessed with this grading scale across phototypes. Moreover, when the data were split by ancestry (White or Caucasian vs. others) statistical analysis of the mean grade changes after 8 weeks did not show statistically significant differences. However, it may still be beneficial to establish a photonumeric scale for darker skin tones in the future, as the aetiology of dark circles, and how their appearance manifests, can vary slightly by skin phototype.

Because of the complex aetiology of dark circles, they can be difficult to treat, especially with cosmetic products. However, in this study we have demonstrated that a cosmetic product, designed to alleviate the symptoms of this condition, reduced the appearance of dark circles in a perceptible and meaningful way.

## Conclusion

To the best of our knowledge, this is the first fully validated photonumeric scale for assessing infraorbital dark circles. The scale is linear, reproducible and repeatable; and sensitive enough to demonstrate changes noticeable to the human eye and is therefore consumer relevant. The scale has also been successfully used in a clinical trial to demonstrate the efficacy of a cosmetic treatment product. We believe that this scale will be a useful tool for clinicians and researchers in this field by providing an easy‐to‐use and sensitive measure of dark circle severity.
